# Context-Dependent Effects of Ranaviral Infection on Northern Leopard Frog Life History Traits

**DOI:** 10.1371/journal.pone.0013723

**Published:** 2010-10-28

**Authors:** Pierre Echaubard, Kevin Little, Bruce Pauli, David Lesbarrères

**Affiliations:** 1 Genetics and Ecology of Amphibians Research Group (GEARG), Department of Biology, Laurentian University, Sudbury, Canada; 2 Environment Canada, Science and Technology Branch, National Wildlife Research Centre, Carleton University, Ottawa, Canada; University of Georgia, United States of America

## Abstract

Pathogens have important effects on host life-history traits, but the magnitude of these effects is often strongly context-dependent. The outcome of an interaction between a host and an infectious agent is often associated with the level of stress experienced by the host. *Ranavirus* causes disease and mortality in amphibian populations in various locations around the world, but most known cases of ranaviral infection have occurred in North America and the United Kingdom. While *Ranavirus* virulence has been investigated, the outcome of *Ranavirus* infection has seldom been related to the host environment. In a factorial experiment, we exposed Northern leopard frog (*Lithobates pipiens*, formerly *Rana pipiens*) tadpoles to different concentrations of *Ranavirus* and investigated the effect of host density on certain life-history traits, namely survival, growth rate, developmental stage and number of days from virus exposure to death. Our results suggest a prominent role of density in driving the direction of the interaction between *L. pipiens* tadpoles and *Ranavirus*. We showed that increasing animal holding density is detrimental for host fitness as mortality rate is higher, day of death earlier, development longer and growth rate significantly lower in high-density tanks. We observed a linear increase of detrimental effects when *Ranavirus* doses increased in low-density conditions, with control tadpoles having a significantly higher overall relative fitness. However, this pattern was no longer observed in high-density conditions, where the effects of increasing *Ranavirus* dose were limited. Infected and control animals fitness were consequently similar. We speculate that the host may eventually diverts the energy required for a metabolic/immune response triggered by the infection (*i.e.*, direct costs of the infection) to better cope with the increase in environmental “stress” associated with high density (*i.e.*, indirect benefits of the infection). Our results illustrate how the net fitness of organisms may be shaped by ecological context and emphasize the necessity of examining the direct/indirect costs and benefits balance to fully understand host-pathogen interactions.

## Introduction

Pathogens are known to affect their hosts in a variety of manners [1]. Most of the studies investigating the relationship between hosts and pathogens have focused on the direct effects that pathogens have on host life history traits, usually including measures such as body length, body weight, growth rate, or survival [2]. Traditionally, quantifying the variation in these traits following infection is used to assess pathogen virulence and host fitness effects. However, beyond the effect a pathogen can have on host-specific fitness traits, attention has also been given to the role that pathogens can play in structuring host communities and affecting population dynamics [3]. While local extinction due to pathogen exposure is rare (see [4] for an example) the extent of detrimental effects caused by a parasite may depend on biological factors such as the pathogen's mode of transmission[5], the host genotype [6], and the host condition [7], [8]. Some of these features have been reported to be highly context dependent. For instance, previous studies have suggested that the degree of differential mortality suffered by infected hosts is linked to the specific host-pathogen relationship, but may also be influenced by the type and level of stress experienced by the host [9].

Relationships between pathogens, parasites and environmental disturbance have recently been addressed in human-modified systems [10], whereby pesticides or other pollutant exposure has typically been found to enhance parasite virulence [11], [12] due to a reduction of the host immune function [13]. At the same time, natural environmental fluctuations can also interact with pathogen virulence. For instance, host population increase may lead to an increase in intraspecific competition for food resources (due to the reduction of per capita food availability) that may affect host traits such as body size, body weight, growth rate, and reproductive ability and in turn affect the pathogen virulence and epidemiology [14]. High density situations may also result in an increase in the contact rate between individuals that can be stressful [15], and may also boost pathogen transmission rate (*i.e.* horizontal transmission [16]) and subsequent pathogen load and virulence. For example the gray treefrog (*Hyla versicolor*) can co-occur in temporary and permanent ponds with a snail (*Pseudosuccinea columella*) that is frequently infected with the digenetic trematode *Telorchis* spp., whose free-swimming cercariae infect *H. versicolor* tadpoles. One study [17] has shown that the presence of infected *P. columella* had strong negative effects on the performance of gray treefrog larvae. This effect, however, depended on whether ponds were temporary or permanent; temporary pond animals were exposed to higher rates of infection, suggesting an important role of snail and tadpole density on subsequent infection status [17]. Density fluctuations may therefore be a key component of host-pathogen interactions, evolution and epidemiology.

*Ranavirus*es are highly virulent pathogens known to infect fish [18], reptiles [19] and a wide range of amphibian species.[20], [21], [22] Effects of *Ranavirus*es seem to be widespread, as they cause disease and mortality at various locations worldwide [23]. Most known cases of ranaviral infection that have been adequately studied have occurred in North America [24], [25], [26] and the UK [27], [28], [29]. *Ranavirus* is now recognized as an important pathogen and Ranaviral disease is acknowledged by the World Organization for Animal Health (OIE) (http://www.oie.int/eng/maladies/en_classification2010.htm?e1d7). This underlies the importance of studying what factors may affect the virulence and distribution of this pathogen.

We aimed to experimentally investigate whether *Ranavirus* effects were modulated by environmental conditions and by inoculation doses. More specifically, we wanted to test whether increased host density would play an important role in influencing the outcome of the interaction between *Ranavirus* and *L. pipiens* tadpoles. In particular, we predicted an increase of *Ranavirus* effects (virulence) when host density and inoculation dose increase. Here, we define virulence as the overall detrimental effect a parasite or pathogen has on the fitness of its host (see [30] for a discussion). Although our results demonstrate a density-condition expression of *Ranavirus* virulence, they only partially support our prediction. We showed that increasing density is detrimental for the host fitness as mortality rate is higher, day of death earlier, development longer and growth rate is significantly lower in high density tanks. However, while we observed a linear increase of virulence when *Ranavirus* doses increased in low density conditions, the pattern disappeared in high density conditions where infected and control individuals had the same relative fitness. Direct costs of infection have potentially been balanced by indirect benefits in deteriorating environmental conditions, therefore sustaining infected host relative fitness.

## Materials and Methods

### 1. The host-pathogen system

#### i. The pathogen: *Ranavirus*

Most of what is presently known about *Ranavirus*es is based on studies of Frog Virus 3 (FV3), and the *Ambistoma tigrinum Virus* (ATV) the type strain of the *Ranavirus* genus for anurans and salamanders respectively [31], [32]. Amphibians are most vulnerable to *Ranavirus* infection during the larval or early metamorphic stages of development, and mortality of infected animals also usually occurs during these developmental stages. While vertical transmission has been suggested [33] but not verified, horizontal transmission of the virus is well known and can occur in three different ways: through direct contact with infected individuals [34], through cannibalism of infected individuals [35], or through exposure to infected water and sediment [24]. Effects of *Ranavirus* infection can sometimes be seen externally as skin ulcerations or systemic haemorrhaging [36]. However signs of infection are not always noticeable [37]. For our study, we used a *Ranavirus* (FV3) isolate derived from the wild type virus originally cultured by Granoff in 1965 [38]. High titer stocks were kindly provided by Dr. Jacques Robert (University of Rochester Medical Center, Rochester, NY, USA).

#### ii. The host: the Northern leopard frog (*Lithobates* (*Rana*) *pipiens*)

In Ontario, Canada, the Northern leopard frog is distributed widely and can be found in a variety of habitats. This species was once quite common through parts of western Canada until declines started occurring during the 1970s [39], [40]. Many populations of Northern leopard frogs have not recovered from these declines [39]. Northern leopard frogs are a good model for the study of *Ranavirus* epidemiology due to their wide distribution, presence with other species potentially acting as reservoirs for pathogens [34], and reported sensitivity to human influence (e.g. pesticide exposure [41]).

### 2. Experimental Procedure

#### i. Experimental design

The tadpoles used in this experiment were obtained from Dr. Vance Trudeau (University of Ottawa, Ottawa, Ontario) in November 2008. These tadpoles were produced from a captive breeding trial of originally wild-caught *L. pipiens* adults that were captured in pristine areas near Ottawa, Ontario. Adult exposure to *Ranavirus* prior to laboratory breeding can not be ruled out. However, there is no evidence for vertical transmission and as all tadpoles were bred from the same parental stock and under the same conditions, there should be no consistent difference between tadpoles used in our experiments. Thirty aquariums containing 3 L of dechlorinated water aged for 3 days were separated into one group of 12 low density tanks and another group of 12 high density tanks composed of the 4 dose treatments (Control, Dose 1, Dose 2. And Dose 3) replicated 3 times. Subsequently, 20 or 40 tadpoles, Gosner stage 25 [42] were randomly added into each of the low or high density tanks, respectively. In our experiment, the low density tanks correspond to a density of 6.6 tadpoles/L while the high density treatment corresponds to a density of 13.3 tadpoles/L. To our knowledge, there are no good data concerning a normal density of *L. pipiens* tadpoles in nature. However, 10 tadpoles/L is commonly used by amphibian rearing facilities to maximize tadpole metamorphosis (Paula Jackman, pers. com.) After 24 hours, all tadpoles from each tank were placed together in a plastic vial along with 100 ml of *Ranavirus-*infected water containing a gradient of *Ranavirus* doses. The four doses of virus were: 100 pfu/ml (Dose 1), 1,000 pfu/ml (Dose 2), 10,000 pfu/ml (Dose 3), plus a control dose (no virus). The tadpoles were left in the “infection solution” for 5 hours before they were transferred, along with the 100 ml of the virus-containing water, back into their respective tanks. Each tank was equipped with an approximately 16 cm long piece of 7.6 cm diameter PVC pipe cut in half to provide some cover for the tadpoles. The tadpoles were fed on a weekly basis with standard tadpole food (Carolina Biological Supply Company, Burlington, NC) at 45 mg/tadpole for week 1, 90 mg/tadpole for week 2, and 180 mg/tadpole for week 3 and for the duration of the experiment. A 12:12 L:D photoperiod was used in all experiments. Prior to *Ranavirus* exposure, 10 tadpoles from each tank were randomly selected to be weighed and their body length (nose to tail) was measured using an electronic caliper (VWR, Catalogue Number 12777.830, ±0.005 mm). This provided an average tadpole size and weight per tank at the beginning of the experiment and was further used to estimate growth rate (see below).

#### ii. Daily monitoring

All tanks were monitored on a daily basis. Dead tadpoles were removed as soon as noticed using assigned disposable plastic pipettes and aquarium nets to avoid any scavenging. Upon removal, dead tadpoles were weighed and their body length and body weight measured as above. The developmental stage of each dead tadpole was recorded [42] and tadpoles were placed into individual plastic vials filled with 70% ethanol and stored at -25°C for subsequent analyses.

Starting on week 3 the water in each tank was replaced once a week by clean filtered water that had been aged for 24 h. As a result, tadpoles were held in virus-containing water for 3 weeks. This was considered long enough for tadpoles to be in close proximity with residual infection therefore approximating natural virus exposure conditions. For instance, *L. clamitans and L. sylvaticus* tadpoles have been reported to show a behavioral response to avoid trematode parasites [43]. It is therefore possible that tadpoles would avoid pathogen-contaminated water, providing relevance to this exposure scenario. Food was administered to each tank after weekly water changes. Removed contaminated water was treated with 5% bleach and left to sit for 2–3 days to kill off any remaining virus before being discarded. The experiment lasted 70 days, which provided enough time for surviving Northern leopard frog tadpoles to metamorphose into juveniles in our controlled laboratory conditions. At the end of the experiment, all the remaining individuals were euthanized using MS-222 following the protocol #2009-03-05 approved by the Laurentian University Animal Care Committee. All the other procedures used in this experiment follow the protocol # 2008-09-03 approved by the Laurentian University Animal Care Committee.

### 3. Life-history traits

In addition to initial body size and weight, final tadpole weight and size were recorded for each tadpole after their death. Percent mortality, average day of mortality, developmental stage and growth rate was also determined. The percent mortality was calculated by determining the percentage of tadpoles that died from each tank at the end of the 70 day experiment. The average day of mortality was calculated as the average day tadpoles died in each tank. The growth rate was calculated for each tadpole by subtracting the average initial tadpole length (calculated from the initial 10 tadpoles measured per tank) from the final tadpole length and dividing by the number of days the tadpole survived. Tadpole developmental stage was assessed using Gosner nomenclature [42].

### 4. Statistical analysis

Data on host fitness traits were analyzed using a full factorial ANOVA model, with density and virus doses as fixed factors. When the standard assumptions of analysis of variance were not met, even after log10 transformation, we used the non-parametric Scheirer-Ray-Hare extension of the Kruskal-Wallis test (*H* statistic; [44]). Sums of squares based on rank transformed data were used. All statistical analyses were performed using JMP software version 8.0.1 (SAS institute Inc., USA).

### 5. Infection screening

Post-experiment screening of infection was done by PCR. Animals were dissected, the liver extracted, crushed into 1.5 ml Eppendorf tubes and the resulting tissue mixture was used in the extraction protocol. DNA was extracted using QIAmp DNeasy Kit following the standard protocol (Qiagen). Extraction negatives, which consisted of lysis buffer and no DNA as well as samples from non-infected individuals, were used to determine if cross-contamination occurred while processing samples [35]. For virus detection, we used a primer known to successfully amplify *Ranavirus*, specifically Frog Virus 3: MCP-*Ranavirus*-F (5′-GACTTGGCCACTTATGAC-3′) and MCP-*Ranavirus*-R (5′- GTCTCTGGAGAAGAAGAA), following the PCR conditions listed in Mao et al [18] and adapted according to [33], [45]: 94°C for 5 min, 94°C for 30 s, 60°C for 30 s and 72°C for 30 s. This was cycled 35 times and completed by a final extension of 2 min at 72°C. This specific primer has been used in other studies [46], [47] and is known to amplify a portion of the major capsid protein within the frog virus 3 genome [18]. Samples are then run on a 1% gel at 100 V for 1 h. Gels were stained with ethidium bromide and virus presence was determined by the presence or absence of a band around 500 base pairs. A sample known to be infected from a previous study was used as a positive control [47]. Overall infection rate for Dose1 was 22%, 25% for Dose 2, 28% for Dose 3. None of the control larvae were infected. In the field, *Ranavirus* infection rates may oscillate between 0 and 63%, but mostly range between 0 and 30% [25], [33]. Our infections rates therefore are in agreement with those found in the field suggesting the transferability of our results to field studies.

## Results

From the 720 tadpoles originally entered into the experiment, 55 individuals were missing due to scavenging/cannibalism and therefore a total of 665 individuals were included in the analysis.

### 1. Percent mortality

**Figure 1:**
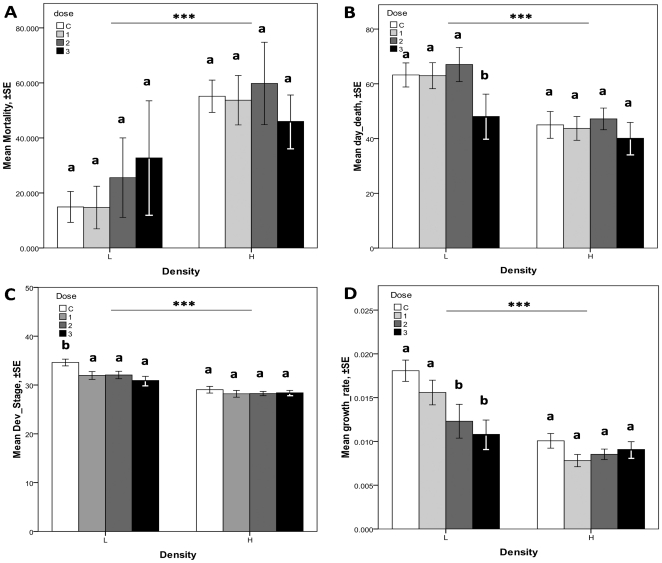
Interactions between tadpole density and exposure dose (Dose 1 =  low dose, Dose 2 =  medium dose, Dose 3 =  high dose; control animals were not exposed to *Ranavirus*). Mortality rate (A), day of death (B) developmental stage (C) and growth rate (D). Letters indicate grouping based on observed means in homogeneous subsets. Significant differences (α = 0.05) between means imply grouping in different subsets (a or b) are based on a Tukey post hoc test.

Few deceased tadpoles showed external signs of *Ranavirus* infection such as blood near the mouth or cloacal region. This observation seems to indicate a rather low virulence of the *Ranavirus* strain used. Nevertheless, the average percent mortality that occurred in the high density tanks was almost twice as high as the percent mortality observed in the low density tanks (Figure 1A, Table S1). In the low density tanks, the high dose (Dose 3) caused the highest percent mortality, followed by Dose 2, then Dose 1, and the control (Figure 1A). The same dose response was not seen in the high density tanks; although the results were not significant, with fewer tadpoles dying when exposed to Dose 3 as compared to tadpoles exposed to lower virus doses.

### 2. Day of Death

The effect of virus dose on the tadpoles' day of mortality was statistically significant (Table S1; *H* = 13.494, df  = 3, p = 0.0037). In low density tanks, tadpoles exposed to Dose 3 died on average on day 46 as compared to tadpoles in the control tanks that died on day 63 on average. Rearing Northern leopard frogs through metamorphosis in the laboratory can be difficult and a certain amount of mortality in control tanks is not unexpected. Density had a significant effect on the day of death for all tanks (Table S1), with tadpoles in the high density tanks dying, on average, earlier than tadpoles in the low density tanks (day 46 and 59 respectively; *H* = 13.125, df  = 1, p = 0.0001). The statistical interaction between dose and density was marginally significant for day of death (*H* = 7.813, df  = 3, p = 0.05; Figure 1B) with tadpoles exposed to Dose 3 dying significantly earlier in low density tanks but not when held in high density conditions (Figure 1B, Table S1).

### 3. Developmental Stage at Death

Overall, tadpoles in our experiment died on average at stage 30 but significant differences were observed between density treatments. In low density treatment tadpoles died on average at stage 32 whereas in high density conditions they died at stage 28 (F = 64.469, df  = 1, p<0.0001, Table S1, Figure 1C). While the statistical interaction between dose and density is not significant (F = 1.763, df  = 3, p = 0.153, Table S1) it is worth noticing that control tadpole in low density tanks reached a significantly more advanced stage of development than infected larvae (F = 4.252, df  =  3, p = 0.006, stage 34 for control *vs.* 32 for dose 1, 32 for dose 2 and 30 for dose 3, Figure 1C).

### 4. Growth Rate

The average growth rate was significantly higher for tadpoles in low density tanks (0.009 g/day *vs*. 0.014 g/day for low and high density tanks, respectively; Table S1, Figure 1D). A statistically significant interaction between density and virus dose (*H* = 12.860, df  = 3, p = 0.0049) was also observed. In the low density tanks the tadpoles with the lowest growth rate were those exposed to the highest virus dose (Figure 1D) indicating a dose response at this density: there were significant differences between Dose 3 and control, and Dose 2 and control (*F* = 14.64, df  = 1, p<0.001 and *F* = 6.07, df  = 1, p = 0.0141, respectively). The difference in growth rate between Dose 1 and control was not statistically significant. In high density tanks however, no statistical differences were observed but tadpoles exposed to the highest dose tended to have a higher growth rate than tadpoles infected by Dose 1 or the controls (Table1, Figure 1D).

## Discussion

In summary, our results revealed that *Ranavirus* virulence is likely density-dependent, and that, when compared to unexposed animals held under the same conditions, the overall effects of *Ranavirus* infection appear to be relatively more severe in animals held in low density as compared to animals held in high density.

### 1. Context-Dependent Virulence of *Ranavirus*

#### i. Doses

In the low density tanks, the effect of FV3 dose on host fitness was consistent with our prediction that an increase in FV3 dose would result in increased mortality, significantly earlier mortality, reduced developmental rate and a significantly decreased growth rate of leopard frog tadpoles (Figure 1). This dose-response effect is supported by a number of previous studies. Duffus et al. [33] showed that an increase in FV3 dose resulted in higher rates of virus infection in wood frog (*L. sylvaticus*) tadpoles. Brunner et al. [37] observed that the odds of mortality increased approximately 2.4 folds for every tenfold increase in *Ambystoma tigrinum* Virus (ATV) dose in tiger salamanders (*Ambystoma tigrinum*), with the greatest mortality at a dose of 10,000 pfu/mL. These authors also observed an earlier day of mortality as ATV dose increased [37]. Our results similarly suggest that an increase in FV3 dose reduces the fitness of leopard frog tadpoles. This is not unexpected as a deterioration of host fitness generally occurs when a pathogen load increases in a host since pathogen multiplication leads to resource depletion in the host potentially leading to death or morbidity if the process is not prevented by host immune defenses [48].

#### ii. Density

In high density tanks, tadpole mortality was higher, day of mortality was earlier, developmental rate was lower and growth rate was lower than in low density tanks (Figure 1 and Table S1). This suggests an overall increase of deleterious effects when population density increases. In our case, the increase in deleterious effects may be explained by at least three mechanisms that may act separately or synergistically: a decrease in resource availability [49], an increase in contact rate, and/or pollution by conspecifics. In our study, tadpoles were fed *ad libitum* to avoid competition for resources and minimize the stress associated with resource appropriation; therefore food availability and stress related to resource appropriation potential should not have been influential in the current experiments. Second, increasing contact rate between individuals can be a stressful situation [15] and may also increase horizontal transmission of pathogens [16]. As a result, the pathogen burden should be higher in individuals in high density conditions, resulting in increased deleterious effects of the pathogen. However, the pattern we observed does not completely support such a scenario. We did observe an overall decrease of host fitness, but the relative fitness of tadpoles that had been exposed to higher doses of *Ranavirus* compared to tadpoles exposed to lower doses of *Ranavirus* do not illustrate a clear effect of dose level on the amount of horizontal transmission (Figure 1). Finally, pollution by conspecifics has been suggested to be important factor in animal health in small aquatic systems [50]. The major nitrogen excretory product of tadpoles is ammonia, a compound which is highly soluble in water. In high density environments, environmental ammonia levels may become toxic to tadpoles. Effects of elevated levels of ammonia include disruptions in cerebral blood flow, interruptions in nerve conductance, modifications in the blood-brain barrier as well as alterations to fat and carbohydrate metabolism in a variety of tissues, potentially resulting in convulsions, coma or death of the organism [51], [52], [53]. While pollution by conspecifics may have been a factor involved in deteriorating tadpole fitness in our experiment, further investigation is needed to disentangle this hypothesis from others.

#### iii. Interaction of dose and density

Both increasing virus doses and host density resulted separately in a deterioration of host fitness. However, the linearity of the relationship between virus dose and host fitness appears to be influenced by the density context in which the infection occurs. In high density tanks tadpoles exposed to the high dose (Dose 3) presented higher survival than tadpoles exposed to lower virus doses or no dose at all, although the results were not significant (Figure 1A). For the time of death, developmental stage and the growth rate in high density tanks, the virus-exposed animals were essentially indistinguishable from the non-exposed animals (Figure 1B–D). On the other hand, there was a trend for a dose-response relationship between virus exposure level and fitness in low density tanks: the higher the dose the more serious were the effects seen in the exposed animals. We propose that for the traits assessed in this experiment, being infected by a pathogen under high density conditions may be relatively less detrimental than expected as its specific effects are masked and diluted by the overall increase of stressful conditions. The relative fitness of infected tadpoles in high density tanks therefore increased as compared to what occurred in animals in low density conditions, in turn leading to a status quo between control and infected individuals in terms of relative fitness. These results suggest a condition-dependence of *Ranavirus* “virulence” in varying density environments whereby *Ranavirus* observed relative virulence decreased as the environment induced more stress in the tadpoles. Several studies support the assumption that environmental stress aggravates the effects of infectious diseases and good examples are given in the context of toxic chemicals [54], malnutrition, thermal stress [55], [56], UV-B radiation [57] and population density increase. However, there are substantial theoretical and empirical reasons to expect that increasing environmental stress does not necessarily lead to increased pathogen virulence [7], [58].

### 2. When Being Infected Is No Longer Detrimental

Our results suggest that for some traits that are directly linked to host fitness (mortality rate, day of death, growth rate), individuals with a substantial pathogen burden are no longer suffering a disadvantage relatively to less- or non-infected individuals in deteriorating or stressful conditions (*e.g.* high density conditions) suggesting limited effects of increasing *Ranavirus* dose in high density conditions. There are several reasons why this may have occurred in the present experiments. First, upon being infected at the beginning of the experiment, the tadpole immune system was likely activated by *Ranavirus* exposure [59] and an associated general metabolic enhancement may have occurred. While amphibian larvae fail to express their MHC Class I immunity until metamorphosis, they do have CD8 T cells [60], and several other immune features are present in the larval immune arsenal early after hatching (see [60], [61]). In *Xenopus laevis* liver, activity of Recombination Activating Genes (RAG) is detectable as early as 3 days after fertilization [62], rearrangement of the Immunoglobulin heavy chain starts on day 5 and the larval type B-Cell Receptor (BCR) and T-Cell Receptor (TCR) repertoires are present within the first week after hatching. While no specific immune response targeting FV3 is likely to have occurred in the larvae (as there is only low or no surface MHC class I expression in tadpoles [61]), it seems nevertheless reasonable to assume that the tadpole's early-stage immune arsenal is activated as a reaction to FV3 infection [63]. Additionally, it is likely that a general metabolic enhancement occurred in response to infection during the average duration of tadpole development. In fact, tadpoles died at stage 30 on average, when independent feeding and normal metabolic functions are already set [42]. Second, we observed a relatively low tadpole mortality rate as compared to similar studies [59]. This suggests a rather low virulence (defined as the detrimental effect on host fitness of a pathogen) of the *Ranavirus* strain used or eventually that the host may have developed some general immunity to this strain in nature. Moreover, the difference in mortality rates observed between our study and other similar studies may be associated with the condition in which the larvae were infected. In our study, we did not inject the tadpoles intraperitonealy with a solution containing FV3 but bath the tadpoles in FV3 solutions to better mimic natural conditions of exposure. It is likely that the amount of viral particles in each individual was therefore lower as compared to intraperitonealy-injected individuals, in turn explaining the relatively low mortality rate observed in our experiment.

Given these three considerations, a potential scenario could be proposed to support the trade-off observed between infection and density stresses. The early activation of the infected tadpoles' immune system, together with an enhancement of their general metabolic state in response to FV3 infection, might have compensated for the detrimental physiological effect of density increases over the experiment. Such interaction could have maintained relatively similar “health” conditions of infected larvae as compared to non-infected larvae under our stressful holding conditions and may therefore reflect a subtle interplay between direct costs, compensatory byproducts (indirect benefits) of infection and stress effects. However, this scenario remains speculative and needs further investigation.

### 3. Conclusions

Our results, in line with theoretical considerations, suggest the importance of considering both direct and indirect effects of pathogen infection in estimating the fitness effects on the host [64]. While further quantitative assessments of factors such as tank pollution and virulence would be needed to better understand the underlying mechanisms of the host-pathogen system we studied under varying density conditions, our results illustrate the importance of considering such context-dependent processes for understanding the dynamics and coevolution of geographically structured populations evolving under different ecological pressures. In the current conceptual framework of the dynamics of host-pathogen evolutionary ecology, these condition-dependent processes need to be integrated by the broad community of pathogen researchers to focus study design and enlarge the scope of investigations. Only by investigating host-pathogen relationships in an integrative framework will researchers truly understand the evolutionary ecology of these relationships [65].

## Supporting Information

Table S1 Results of analysis of variance (F ratio) and Sherrer-Ray-Hare extension of the Kruskal-Wallis test (H ratio) representing the effect of dose, density as fixed effects and their interaction on percent mortality (% mortality), day of death, developmental stage (Dev. Stage; [40]) and growth rate of leopard frog tadpoles. All tadpoles were included to calculate percent mortality and day of death, but only tadpoles that survived until the end of the experiment were used to calculate growth rate. * indicates significance (p<0.05). For each dependent/independent variable pair, corresponding values are given: d1 = dose 1, d2 = dose 2, d3 = dose 3, c =  control; h  =  high density, l =  low density; L1  =  low-density dose 1, L2 =  low-density dose 2, L3 =  low-density dose 3, Lc =  low-density control, H1 =  high-density dose 1, H2 =  high-density dose 2, H3 =  high-density dose 3, Hc =  high-density control.
(0.01 MB PDF)
