# Supplementary material for: Context-Dependent Effects of Ranaviral Infection on Northern Leopard Frog Life History Traits
Source: PLoS One. 2010 Oct 28;5(10):e13723. doi: 10.1371/journal.pone.0013723 (PMC2965661; doi:10.1371/journal.pone.0013723)
Supplement: Table S1 — Results of analysis of variance (F ratio) and Sherrer-Ray-Hare extension of the Kruskal-Wallis test (H ratio) representing the effect of dose, density as fixed effects and their interaction on percent mortality (% mortality), day of death, developmental stage (Dev. Stage; [40]) and growth rate of leopard frog tadpoles. All tadpoles were included to calculate percent mortality and day of death, but only tadpoles that survived until the end of the experiment were used to calculate growth rate. * indicates significance (p<0.05). For each dependent/independent variable pair, corresponding values are given: d1 = dose 1, d2 = dose 2, d3 = dose 3, c = control; h = high density, l = low density; L1 = low-density dose 1, L2 = low-density dose 2, L3 = low-density dose 3, Lc = low-density control, H1 = high-density dose 1, H2 = high-density dose 2, H3 = high-density dose 3, Hc = high-density control. (0.01 MB PDF) [file pone.0013723.s001.pdf]

**Responses**/Effects	***Raw numbers***	***F/H* Ratio**	**Df**	**p value**
**%Mortality**				
Dose	*d1=34.20, d2=48.38, d3=38.41,c=35.03*	$F_{3, 624} = 0.150$	3	0.9281
Density	*h=42.45, l=35.23*	$F_{1, 624} = 10.175$	1	0.0054*
Dose x	*L1= 14.69, L2= 25.55, L3= 32.73,Lc= 14.91, H1= 53.71, H2= 59.79, H3= 46, Hc= 55.15*	$F_{3, 624} = 0.438$	3	0.729
**Day of Death**				
Dose	*d1=50.42, d2=49.14 ,d3=42.58, c=51.28*	*H*= 13.494	3	0.0037*
Density	*h=46,l=59*	*H*= 13.125	1	0.0003*
Dose x	*L1=62.96, L2=63.38, L3=48.05, Lc=63.23, H1=43.7, H2=47.18, H3=40.18, Hc=44.99*	*H*= 7.813	3	0.05*
**Dev. Stage**				
Dose	*d1=29.93, d2=29.10, d3=29.19, c=31.348*	$F_{3, 624} = 4.008$	3	0.008*
Density	*h=28.47, l=32.382*	$F1_{, 624} = 64.469$	1	0.0001*
Dose x	*L1=31.9, L2=32, L3=30.9, Lc=34.6, H1=28.2, H2=28.2, H3=28.4, Hc=29*	$F_{3, 624} = 1.763$	3	0.153*
**Growth Rate**				
Dose	*d1=0.010,d2=0.009,d3=0.009,c=0.012*	*H* = 14.196	3	0.0027*
Density	*h=0.009,l=*0.014	*H* = 39.252	1	<0.0001*
Dose x	*L1= 0.015, L2= 0.012, L3= 0.010, Lc= 0.018, H1= 0.007, H2= 0.008, H3= 0.009, Hc= 0.010*	*H* = 12.860	3	0.0049*
